# Extracts of endophytic fungi from leaves of selected Nigerian ethnomedicinal plants exhibited antioxidant activity

**DOI:** 10.1186/s12906-021-03269-3

**Published:** 2021-03-20

**Authors:** Mutiat Ibrahim, Elizabeth Oyebanji, Muinah Fowora, Ayobami Aiyeolemi, Chiamaka Orabuchi, Babajide Akinnawo, Adedotun A. Adekunle

**Affiliations:** 1 Department of Pharmacognosy, Faculty of Pharmacy, University of Lagos, College of Medicine campus, Idi-Araba, Lagos state, Nigeria; 2 Department of Biological Sciences, Mountain Top University, Magboro, Ogun State Nigeria; 3 Molecular Biology and Biotechnology Department, Nigeria Institute of Medical Research (NIMR), Yaba, Lagos state, Nigeria; 4 Department of Botany, Faculty of Science, University of Lagos, Akoka, Lagos state, Nigeria

**Keywords:** Nigerian plants, Endophytic fungi, Ethnomedicinal plants, ITS-rDNA, Free radical scavenging, Secondary metabolites

## Abstract

**Background:**

Plants with an ethnobotanical history are known to harbor diverse group of endophytic fungi, which constitute major natural sources of bioactive compounds. In the present study, we evaluated the antioxidant activity of endophytic fungi from eight Nigerian ethnomedicinal plants. Endophytic fungi were isolated from the leaves of *Acalypha ornata*, *Albizia zygia*, *Alchornea cordifolia*, *Chrysophyllum albidum*, *Ficus exasperata*, *Gomphrena celosioides*, *Millettia thonningii*, and *Newbouldia laevis*.

**Methods:**

Endophytic fungi were isolated from the leaves of selected plants via surface sterilization. Isolated fungi were identified by internal transcribed spacer (ITS-rDNA) sequence analysis. Pure fungal strains were subjected to fermentation process on solid rice medium and metabolites extracted using ethyl-acetate. Fungal crude extracts were screened for antioxidant activity using 2, 2- diphenyl-1-picrylhydrazyl (DPPH) radical scavenging and reduction of ferric ion assays. Gas chromatography/mass spectrometry (GC/MS) analysis was used to identify the major chemical constituents in active fungal extracts.

**Results:**

A total of eighteen fungal endophytes with fungal codes CU (061 and 062); ZA (161, 162, 163, and 164); LO (261); CA (041, 042, and 043); FE (081, 082, and 084); GE (091); MO (211 and 212); and NA (021 and 022) were isolated from the eight ethnomedicinal plants *A. ornata*, *A. zygia*, *A. cordifolia*, *C. albidum*, *F*. *exasperata*, *G. celosioides*, *M*. *thonningii*, and *N. laevis* respectively. ZA 163 and MO 211 fungal extracts showed significant (*p* < 0.05) radical scavenging activity with IC_50_ values of 50.53 ± 0.01 and 86.69 ± 0.02 μg/ml respectively. Fungal extract CA 041 demonstrated significantly (*p* < 0.01) higher iron chelating activity than standard gallic acid with absorbance values of 0.803 and 1.107 at 250 and 500 μg/ml concentrations respectively. Pyrogallol, phenol, 2,6-dimethoxy-, phytol, dl-alpha-tocopherol, alpha-tocospiro, oleamide, methyl stearate, oleic acid, palmitic acid, campesterol, stigmasterol, β-sitosterol, urs-12-en-24-oic acid, 3-oxo-, methyl ester, lup-20(29)-en-3-one, and lupeol were detected in the selected active extracts.

**Conclusion:**

These results showed that leaves of the selected Nigerian plants harbor diverse group of endophytic fungi, which can be potential antioxidant resource.

**Graphical abstract:**

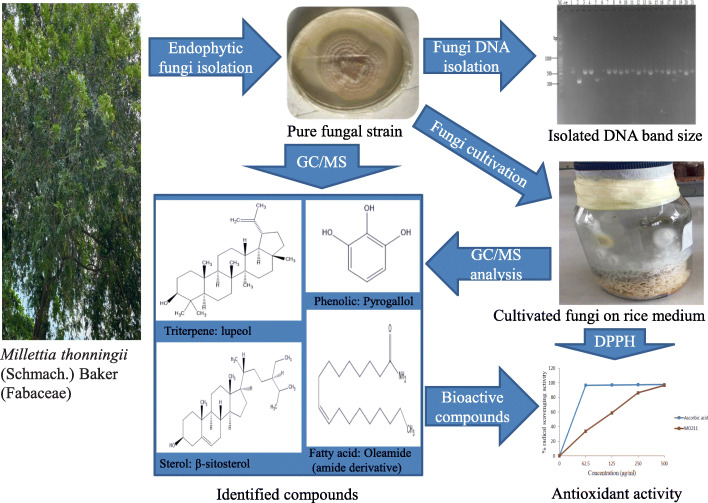

## Background

Endophytes are microorganisms that reside within plant tissues and do not cause any deleterious effect to the plants. These microorganisms consist of bacterial and fungal communities that colonize and spend the whole or part of their life cycle inside tissues of the host, without causing noticeable symptoms of plant diseases [1, 2]. Endophytic fungi are found in almost plant parts and act as chemical synthesizers inside the host plant, producing a wide range of bioactive secondary metabolites [3, 4].

Endophytic fungi from the genera *Colletotrichum*, *Fusarium*, *Alternaria* and *Aspergillus*, isolated from medicinally important plants exhibit a variety of biological activities such as anticancer, antimicrobial, antifungal, immunomodulatory, antitubercular, and antioxidant activities with wide application in agrochemical and pharmaceutical industries [5–8]. These biological activities demonstrated by endophytes have been attributed to isolated and identified secondary metabolites such as alkaloids, terpenoids, steroids, quinones, isocoumarin derivatives, flavanoids, peptides, and phenols present in the fungal extracts [9–14]. Therefore, exploring endophytic fungi that reside in medicinal plant species would provide vast opportunities to discover new medicinally important metabolites [15].

Production of reactive oxygen species known as free radicals occurs frequently in all cells as part of normal metabolic process. However, free radicals have been implicated in the pathogenesis of quite a number of chronic diseases such as diabetes, neurological disorders, and cancer [16]. Free radical scavenging property of antioxidant agents inhibits or delays cellular damage. Dietary intake as well as adequate amount of exogenous antioxidants would prevent the pathological conditions induced by free radicals [17]. However, the use of synthesized antioxidants in the prevention of free radical damage may exude toxic side effects thus the search for natural antioxidant agents is required [18, 19]. Endophytic fungi can synthesize secondary metabolites with antioxidant activity that can interrupt the chain reaction of reactive oxygen species [20, 21].

Medicinal plants growing in natural habitats are promising hosts of endophytic fungi that might produce bioactive secondary metabolites of pharmaceutical relevance. Different plant parts, especially the leaves, stems, and roots are considered an enormous repository of these fungal endophytes with reported cytotoxic, antifungal, antiviral, and antimicrobial activities [22–24]. The genetic resources of these plants found in Nigeria are a veritable source of pharmaceuticals and therapeutics with significant information on their phytochemistry. Additionally, these plants are used locally in the treatment of various diseases. Hence, this study was carried out to isolate and screen endophytic fungi with antioxidant activity from eight selected ethnomedicinal plants namely: *Acalypha ornata* Hochst. ex A. Rich (Euphorbiaceae), *Albizia zygia* (DC.) J.F. Macbr. (Fabaceae), *Alchornea cordifolia* (Schum. & Thonn.) Müll-Arg (Euphorbiaceae), *Chrysophyllum albidum* G. Don (Sapotaceae), *Ficus exasperata* Vahl (Moraceae), *Gomphrena celosioides* Mart. (Amaranthaceae), *Millettia thonningii* (Schmach.) Baker (Fabaceae), and *Newbouldia laevis* (P. Beauv.) Seem. ex Bureau (Bignoniaceae).

## Methods

### Collection of plant samples and identification

Fresh and healthy leaf samples of *Acalypha ornata* Hochst. ex A. Rich, *Albizia zygia* (DC.) J.F. Macbr., *Alchornea cordifolia* (Schum. & Thonn.) Müll-Arg, *Chrysophyllum albidum* G. Don, *Ficus exasperata* Vahl, *Gomphrena celosioides* Mart., *Millettia thonningii* (Schmach.) Baker, and *Newbouldia laevis* (P. Beauv.) Seem. ex Bureau were collected within the main campus of the University of Lagos, Lagos State, Nigeria in the month of March, 2019. The plant samples were identified and authenticated by Dr. George I. Nodza at the Herbarium of the Department of Botany, University of Lagos where herbarium specimen and voucher specimen number (LUH 8256–62; 8264) for each sample was deposited. Each sample was kept separately in a sealed plastic bag and brought to the laboratory on the same day for the isolation of fungal endophytes.

### Isolation of endophytic fungi

The collected plant samples were surface sterilized according to the method previously described [25, 26] with modifications. To remove dust and debris, samples were washed thoroughly under running tap water, followed by washing in distilled water. The leaf samples were cut into small segments measuring 2–4 mm using sterile razor blades. Surface sterilization of leaf segments was carried out by sequential immersion in 70% ethanol for 60 s, 0.5% sodium hypochlorite for 5 min, 70% ethanol for 30 s, followed by a final rinse in sterilized distilled water for 5 min. Sterilized leaf segments were air-dried aseptically and placed on petri dishes containing potato dextrose agar amended with 0.01% gentamycin antibiotic for the direct contact of cut edges with the nutrient media. Three segments of each plant sample were placed on PDA plates, sealed with parafilm and incubated at 27 °C for 5–7 days. Samples were checked daily for visual growth of fungi.

### Purification and preservation of endophytic fungi

Following the incubation period, different fungal strains emerged from each sample and individual strains were isolated by transferring hyphal tips onto a fresh PDA medium. This process was repeated several times until a pure endophytic fungal strain with uniform colony was obtained. Pure endophytic fungi isolates were transferred separately to PDA slants and 15% (v/v) sterilized glycerol solution. Growth of endophytic fungi strains in both media was observed for 3–5 days. Glycerol stock solution and PDA slants were maintained at − 20 °C and 4 °C respectively till further use.

### Molecular identification

Identification of endophytic fungi was performed using molecular characterization and sequencing of the internal transcribed spacer region (ITS).

#### Endophytic fungi DNA extraction

Deoxyribonucleic acid (DNA) extraction of fungal strains was done using Zymo fungal / bacteria DNA extraction kit (Zymo Research Corp., South Africa) according to the manufacturer’s instructions. Briefly, pure endophytic fungi strains isolated from the plant samples were grown in potato dextrose broth (PDB) for 6–7 days. Following the formation of endophytes colony, fungal mycelium was drained and homogenized in 200 μL of phosphate buffered saline to aid lysis. About 50 mg of homogenate was harvested into a 1.5 ml microcentrifuge tube and 750 μL lysis solution was added to the tube. The tube was vortex vigorously for 30–60 s and 400 μL of supernatant was transferred into a Zymo-Spin™ IV spin filter in a collection tube and centrifuged at 10,000 rpm for 1 min. After centrifugation, 1200 μL of Fungal/Bacterial DNA binding buffer was added to filtrate in the collection tube. 800 μL of the mixture was then transferred to a Zymo-Spin™ IIC column in a collection tube. The reaction mixture was centrifuged at 10,000 rpm for 1 min. The flow through was discarded and the remaining mixture was centrifuged again at 10,000 rpm for 1 min in the same tube. The column was pre-washed with 200 μL DNA Pre-Wash buffer and centrifuged at 10,000 rpm for 1 min. This was followed by a wash with 500 μL Fungal/Bacterial DNA Wash Buffer and centrifuged at 10,000 rpm for 1 min. The DNA was eluted from the Zymo-Spin™ IIC column to a sterile 1.5 ml microcentrifuge tube using 100 μL of DNA Elution Buffer. The purity and concentration of extracted DNA was evaluated using a NANODROP (ND 1000) spectrophotometer (Thermo Scientific, USA).

#### Amplification and sequencing

Polymerase chain reaction was carried out to amplify the ITS gene of specific DNA of each fungus using the primer pair ITS-1 (5′-TCCGTAGGTGAACCTGCGG) and ITS-4 (5′-TCCTCCGCTTATTGATATGC) as previously described [27, 28]. PCR was done using the Solis Biodyne 5x Hot FIREPol® Master Mix Ready to Load. PCR reaction was performed in a total volume of 20 μL containing 1 x blend master mix buffer, 1.5 mM MgCl_2_, 200 μM of each deoxynucleoside triphosphate (dNTP), 25 pmol ITS1 and ITS4, 2 units of hot FIREPol DNA polymerase and 10–200 ng of DNA. Sterile distilled water was used to make up the reaction mixture. PCR amplification was performed in PTC 100 Peltier Thermal Cycler (MJ Research) using the following protocols: denaturation at 95 °C for 15 min; 35 amplification cycles at 95 °C for 30 s; primer-specific annealing at 58 °C for 1 min and elongation at 72 °C for 90 s; a final elongation at 72 °C for 10 min. Amplified DNA was checked by electrophoresis at 80 V for 90 min in a 1.5% agarose gel stained with ethidium bromide. The band to PCR product was visualized with a photodocumentation system (Cleaver Scientific, UK).

The amplified products were purified with exo sap and sequenced by Epoch Life science, USA using Sanger sequencing method. Identification of endophytic fungi was performed on the basis of similarity of amplified sequence with ITS sequence data from strains available in the US National Centre for Biotechnology Information (NCBI) database using Basic Local Alignment Search Tool (BLAST) N sequence match routines.

### Fermentation and extraction of isolated fungal metabolites

Cultivation of isolated fungal endophytes was carried out in a conical flask containing sterilized rice medium as previously described [25, 29] with modifications. Approximately 200 g of rice was soaked in 200 ml sterile water for 10 min in a glass bottle. The flask with its content was autoclaved at 121 °C for 20 min and allowed to cool. Three to four pieces of pure mycelia agar plugs were inoculated aseptically into the cooled solid rice medium. A flask of autoclaved solid rice medium without inoculum served as the control. Fungal strain was allowed to grow on rice medium at room temperature (27 ± 2 °C) for 4–6 weeks with a routine check. After the incubation period, about 500 ml of ethyl-acetate was added into the culture flask and left overnight at room temperature. The crude ethyl-acetate fungal mixture was filtered through a Whatman filter paper No. 1 under vacuum using a Buchner funnel. Collected supernatant was evaporated to dryness under vacuum on a rotary evaporator (Buchi, Switzerland) at 40 °C to obtain crude fungal extract.

### In vitro antioxidant activity of fungal extracts

#### DPPH radical scavenging activity

Free radical scavenging activities of fungal extracts were measured using 2, 2-diphenyl-1-picryl-hydrazyl (DPPH) as previously described [30, 31]. Briefly, 2 ml of varying concentrations (62.5–500 μg/ml) of fungal extracts and positive standard (ascorbic acid) prepared in methanol was mixed with 2 ml of 0.135 mM DPPH in methanol. The mixture was shaken vigorously and incubated in the dark at room temperature for 30 min. Mixture of methanol and DPPH without fungal extract served as the control. Absorbance of samples was measured at 517 nm against the blank tube that contains methanol and used to maintain zero of the UV-vis spectrophotometer. The assay was carried out in three replicates. Inhibition of DPPH was calculated as a percentage of radical scavenging activity of each fungal extract using the formula:

Percentage of radical scavenging activity = [(A_control_ - A_sample_)/A_control_] × 100.

Where A is the absorbance reading. IC_50_ value (μg/mL) was calculated from percentage of radical scavenging activity against extract concentrations.

#### Reduction of Fe^3+^ ions by ortho-phenanthroline

*O*-phenanthroline assay was used to determine the reducing capacity of fungal extracts as previously described [32, 33]. A reaction mixture containing 1 ml of 2 mM phenanthroline, 2 ml of 0.2 mM ferric chloride hexahydrate (FeCl_3_.6H_2_O), and 2 ml of different concentrations (62.5–500 μg/ml) of fungal extracts was shaken and incubated at ambient temperature for 10 min. Absorbance was measured at 510 nm using a UV-vis spectrophotometer. Gallic acid served as positive standard.

### Identification of bioactive constituents by GC/MS

Gas chromatography/mass spectrometry (GC/MS) analysis of bioactive fungal extracts was performed using Agilent 7820A gas chromatograph coupled to an Agilent 5975C inert mass selective detector (MSD) with triple axis detector operated in an electron impact (EI) mode with ionization energy of 70 eV. An HP-5 capillary column coated with 5% phenyl methyl siloxane (30 m × 0.32 mm diameter × 0.25 μm film thickness) was used for the separation. Sample preparation was performed according to the procedure described [34]. The sample (1 μL, diluted 1: 100 in dichloromethane) was injected in splitless mode at an injection temperature of 300 °C. Purge flow to split vent was 15 ml/min at 0.75 min with a total flow of 16.654 ml/min. Helium was used as the carrier gas at the flow rate of 1.487 ml/min with initial nominal pressure of 1.4902 psi and an average velocity of 44.22 cm/sec. The oven temperature was initially programmed at 40 °C for 1 min then ramped at 12 °C / min to 300 °C for 10 min. Run time was 32.667 min with a hold time of 5 °C / min. Identification of the chromatographic peaks was based on comparisons of their relative retention times and mass spectra with those obtained from the NIST14.L library data.

### Statistical analysis

All assays were carried out in three replicates and results are expressed as mean ± standard error of mean (SEM). IC_50_ values were obtained by interpolations from standard curves. Data were analyzed using Graph Pad Prism 8.

## Results

### Isolation of endophytic fungi

**Fig. 1:**
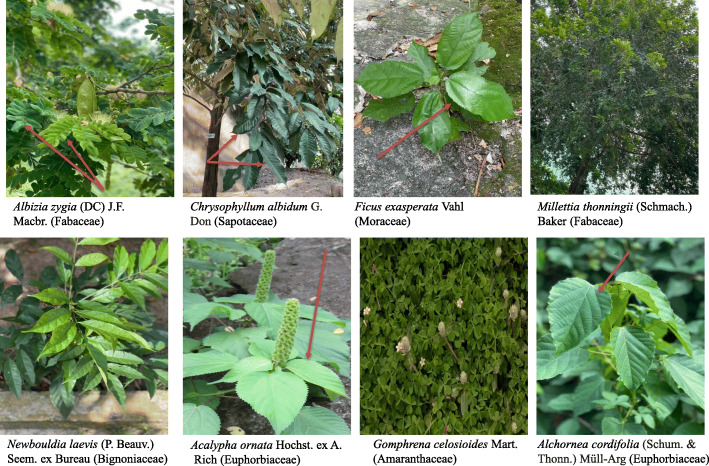
Pictures of selected plants from University of Lagos, main campus, Nigeria used for fungal isolation

**Fig. 2:**
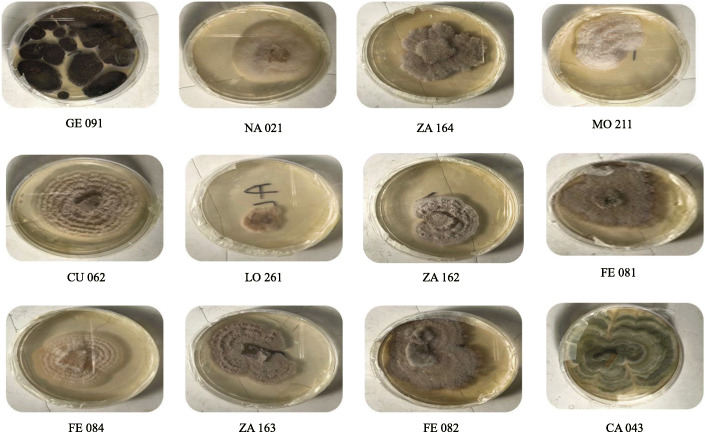
Pictures of isolated endophytic fungi species on culture plate

Eighteen pure fungal endophytes were successfully isolated from surface sterilized fresh leaf samples of eight medicinal plants: *Acalypha ornata*, *Albizia zygia*, *Alchornea cordifolia*, *Chrysophyllum albidum*, *Ficus exasperata*, *Gomphrena celosioides*, *Millettia thonningii*, and *Newbouldia laevis* (Fig. 1). The leaves of *A. zygia* were found to host the highest number of endophytic fungi with four isolates: ZA 161, ZA 162, ZA 163, and ZA 164 (Table 1). This was followed by the leaves of *C. albidum* with three isolates: CA 041, CA 042, and CA 043 and *F. exasperata* with three isolates: FE 081, FE 082, and FE 083 (Table 1). Two endophytic fungal isolates were obtained from the leaves of *A. ornata* (CU 061 and CU 062); *M. thonningii* (MO 211 and MO 212); *N. laevis* (NA 021 and NA 022). *A. cordifolia* and *G. celosioides* leaf samples hosted one fungal isolate LO 261 and GE 091 respectively (Table 1). Pictorial representation of isolated endophytic fungi is as shown in Fig. 2.

**Table 1:** Identification of endophytic fungi isolated from selected ethnomedicinal plants from University of Lagos, Nigeria

S/N	Plant source	Voucher specimen number	Number of isolated endophytic fungi	Fungal code
1.	*Albizia zygia* (DC.) J.F. Macbr. (Fabaceae)	LUH 8256	4	ZA 161
				ZA 162
				ZA 163
				ZA 164
2.	*Chrysophyllum albidum* G. Don (Sapotaceae)	LUH 8262	3	CA 041
				CA 042
				CA 043
3.	*Ficus exasperata* Vahl (Moraceae)	LUH 8264	3	FE 081
				FE 082
				FE 084
4.	*Acalypha ornata* Hochst. ex A. Rich (Euphorbiaceae)	LUH 8260	2	CU 061
				CU 062
5.	*Millettia thonningii* (Schmach.) Baker (Fabaceae)	LUH 8261	2	MO 211
				MO 212
6.	*Newbouldia laevis* (P. Beauv.) Seem. ex Bureau (Bignoniaceae)	LUH 8257	2	NA 021
				NA 022
7.	*Alchornea cordifolia* (Schum. & Thonn.) Müll-Arg (Euphorbiaceae)	LUH 8258	1	LO 261
8.	*Gomphrena celosioides* Mart. (Amaranthaceae)	LUH 8259	1	GE 091

### Extraction of fungal metabolites

All the fungi isolates were cultivated on solid media and extracted with ethyl-acetate to afford fungal extracts of varying yield, which range from 44 to 2002 mg per 200 g rice medium (Table 2).

**Table 2:** Yield of fungal extracts and reduction in ferric ion activity of standard and endophytic fungi

S/N	Standard and Fungal code	Yield of endophytic fungi extract (mg) in 200 g of rice medium	Absorbance readings at varying concentrations (μg/ml)			
			62.5	125	250	500
1.	Gallic acid	–	0.479 ± 0.011	0.486 ± 0.004	0.499 ± 0.009	0.521 ± 0.004
2.	ZA 161	0233	0.277 ± 0.001*	0.302 ± 0.002*	0.333 ± 0.005*	0.460 ± 0.009*
3.	ZA 162	1390	0.212 ± 0.017*	0.240 ± 0.003*	0.233 ± 0.021*	0.453 ± 0.086
4.	ZA 163	0044	0.304 ± 0.002*	0.310 ± 0.005*	0.424 ± 0.009*	1.190 ± 0.001*
5.	ZA 164	0080	0.219 ± 0.005*	0.240 ± 0.006*	0.278 ± 0.006*	0.324 ± 0.006*
6.	CA 041	0900	0.296 ± 0.005*	0.412 ± 0.009*	0.843 ± 0.011*	1.070 ± 0.038*
7.	CA 042	1280	0.230 ± 0.003*	0.238 ± 0.005*	0.453 ± 0.118	0.932 ± 0.002*
8.	CA 043	0930	0.233 ± 0.004*	0.256 ± 0.005*	0.478 ± 0.047	0.994 ± 0.002*
9.	FE 081	1650	0.219 ± 0.007*	0.249 ± 0.005*	0.280 ± 0.002*	0.387 ± 0.011*
10.	FE 082	1247	0.300 ± 0.012*	0.320 ± 0.002*	0.382 ± 0.021*	0.852 ± 0.008*
11.	FE 084	1960	0.239 ± 0.010*	0.245 ± 0.005*	0.336 ± 0.011*	0.869 ± 0.064*
12.	CU 061	2002	0.428 ± 0.046	0.357 ± 0.010*	0.249 ± 0.004*	0.142 ± 0.003*
13.	CU 062	0066	0.452 ± 0.018	0.472 ± 0.007	0.316 ± 0.048	0.179 ± 0.019*
14.	MO 211	0044	0.307 ± 0.007*	0.341 ± 0.012*	0.351 ± 0.004*	0.440 ± 0.007*
15.	MO 212	1402	0.236 ± 0.004*	0.272 ± 0.007*	0.356 ± 0.034	0.441 ± 0.021
16.	NA 021	0539	0.224 ± 0.007*	0.251 ± 0.004*	0.252 ± 0.020*	0.301 ± 0.005*
17.	NA 022	0166	0.263 ± 0.001*	0.330 ± 0.008*	0.367 ± 0.004*	0.462 ± 0.003*
18.	LO 261	1149	0.259 ± 0.005*	0.332 ± 0.005*	0.490 ± 0.006	0.803 ± 0.011*
19.	GE 091	1010	0.352 ± 0.002*	0.400 ± 0.003*	0.640 ± 0.024*	0.977 ± 0.009*

Absorbance readings are expressed as mean ± SEM. * *p* < 0.01 significant difference between concentrations of same extract

### Antioxidant activity of fungal extracts

#### DPPH radical scavenging activity

**Fig. 3:**
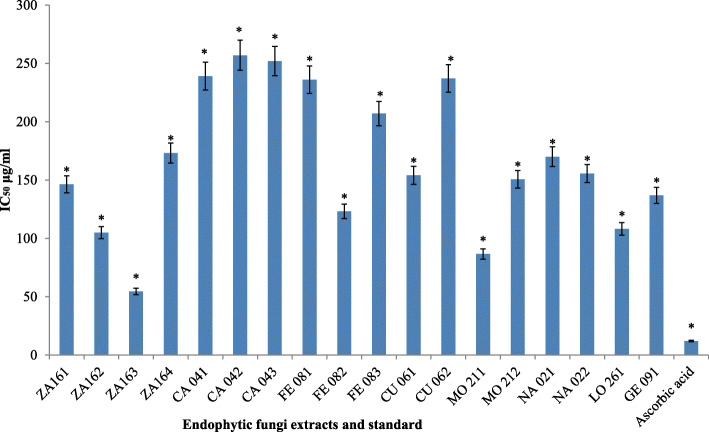
IC_50_ values of standard and extracts of endophytic fungi in DPPH radical scavenging assay.* *p* < 0.05 significant difference between concentrations of same extract

Figure 3 showed the IC_50_ values of fungal extracts, which was used to indicate antioxidant capacity. Extracts of all endophytic fungal isolates exhibited significant (*p* < 0.05) radical scavenging activity with an IC_50_ value range of 50.53 μg/ml to 257.0 μg/ml. The standard ascorbic acid revealed a value of 12.07 μg/ml. The highest antioxidant activity with an IC_50_ value of 50.53 ± 0.01 μg/ml was displayed by extract of endophytic fungi ZA163 isolated from the leaves of *Albizia zygia*. This was followed by extract of fungal isolate MO211 obtained from the leaves of *Millettia thonningii* with an IC_50_ value of 86.69 ± 0.02 μg/ml. Extracts of fungal isolates CA 041, CA 043, and CA 042 isolated from *Chrysophyllum albidum* leaves showed the least radical scavenging activity with IC_50_ values of 239.20 ± 0.04, 252.0 ± 0.11 and 257.0 ± 0.11 μg/ml respectively.

#### Reduction of Fe^3+^ ions by ortho-phenanthroline

Except for extracts of endophytic fungi CU 061 and CU 062 isolated from *Acalypha ornata*, all fungal extracts showed a dose dependent antioxidant activity with an increase in absorbance value as concentration increases (Table 2). At 250 and 500 μg/ml concentrations, fungal extracts CA 041 from *Chrysophyllum albidum* and GE 091 from *Gomphrena celosioides* demonstrated significantly (*p* < 0.01) higher antioxidant activity (0.843 and 1.070; 0.640 and 0.977 respectively) than standard gallic acid (0.499 and 0.521). Also at 500 μg/ml concentration, the absorbance values of fungal extracts ZA 163 (1.190) from *Albizia zygia*; LO 261 (0.803) from *Alchornea cordifolia*; CA 042 (0.932) and CA 043 (0.994) from *C. albidum*; FE 084 (0.869) and FE 082 (0.852) from *Ficus exasperata*; demonstrated significant (*p* < 0.01) higher antioxidant capability than gallic acid (Table 2).

### Identification of bioactive constituents by GC/MS

**Fig. 4:**
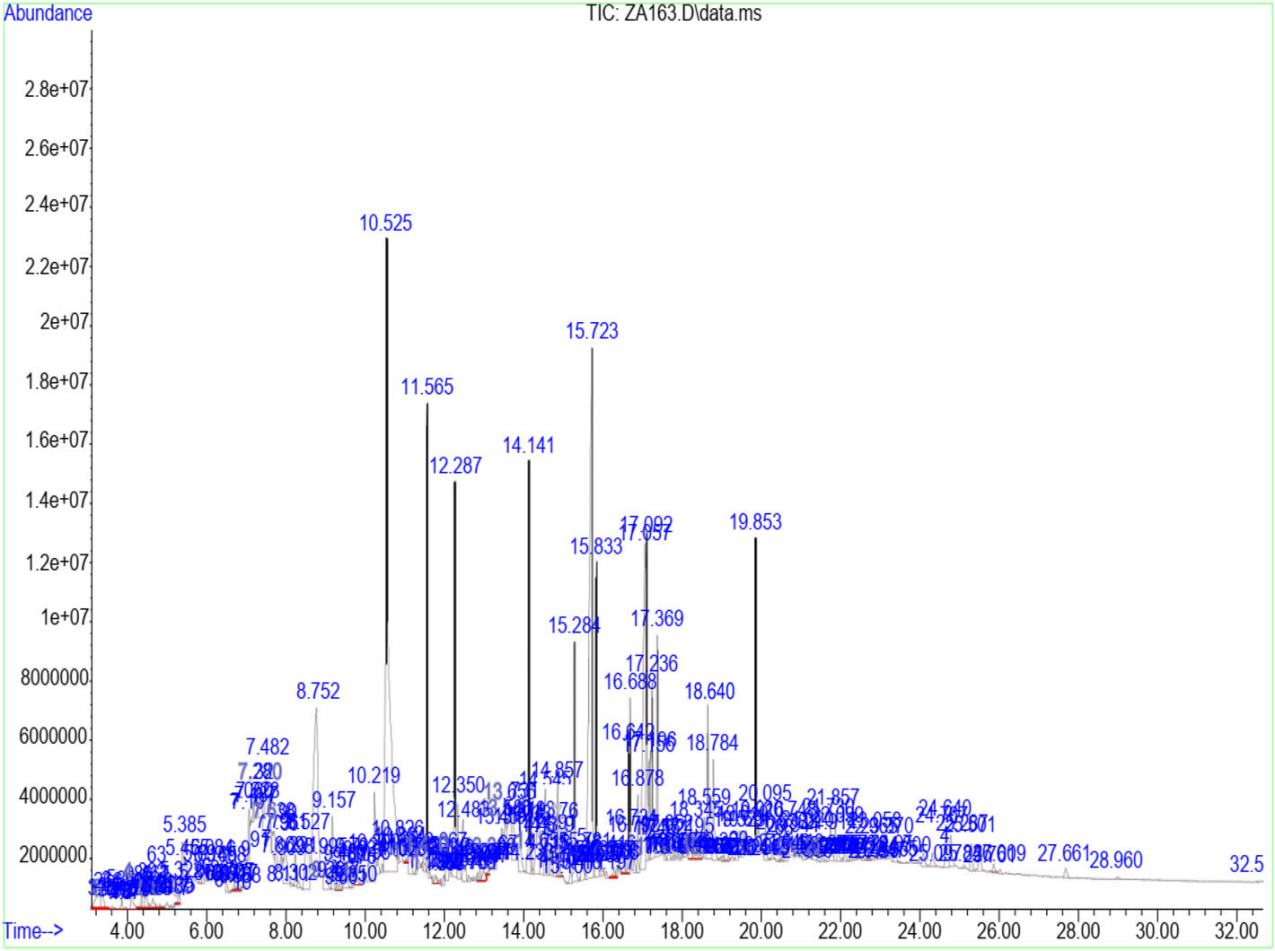
GC/MS chromatogram of fungal extract ZA163

**Fig. 5:**
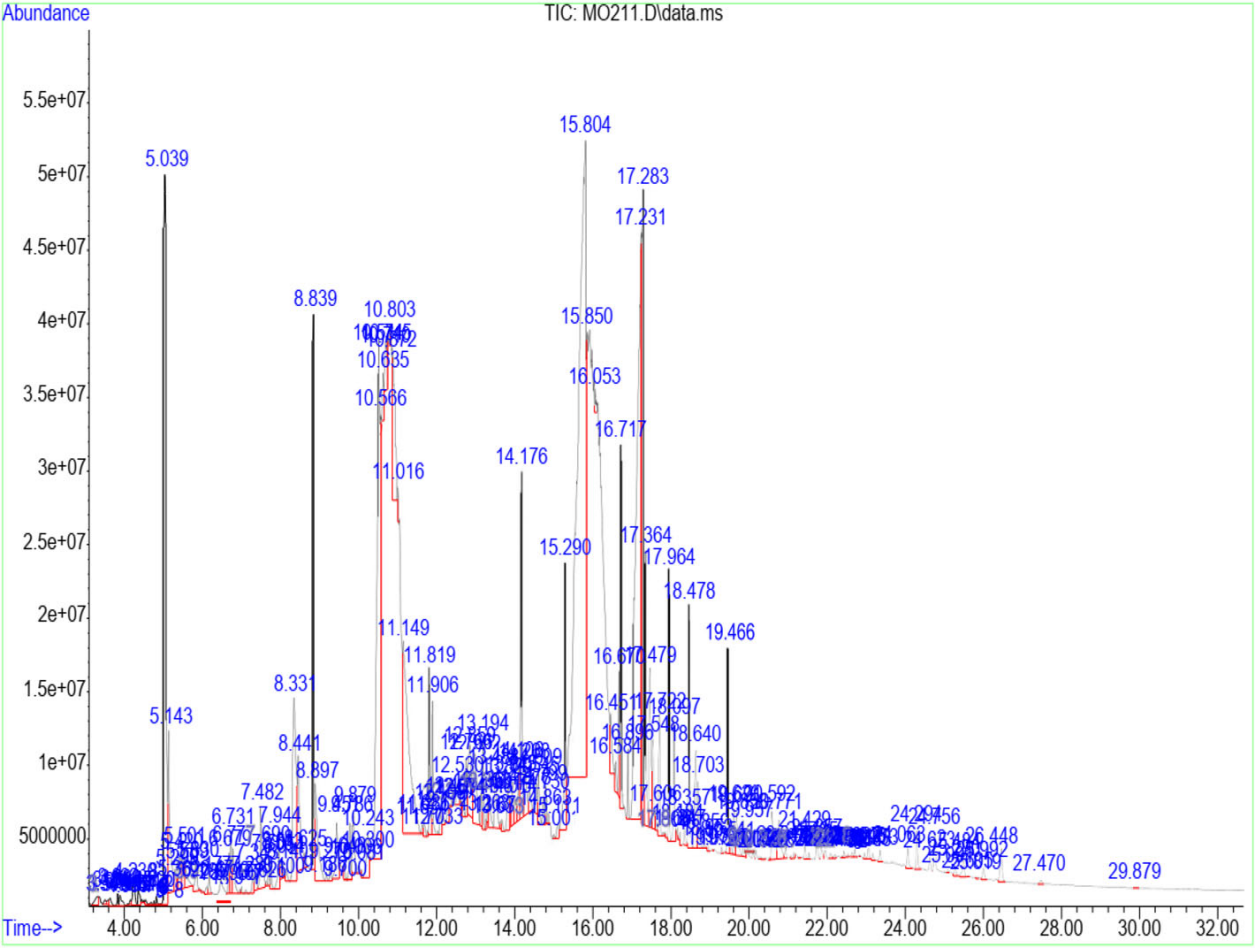
GC/MS chromatogram of fungal extract MO211

**Fig. 6:**
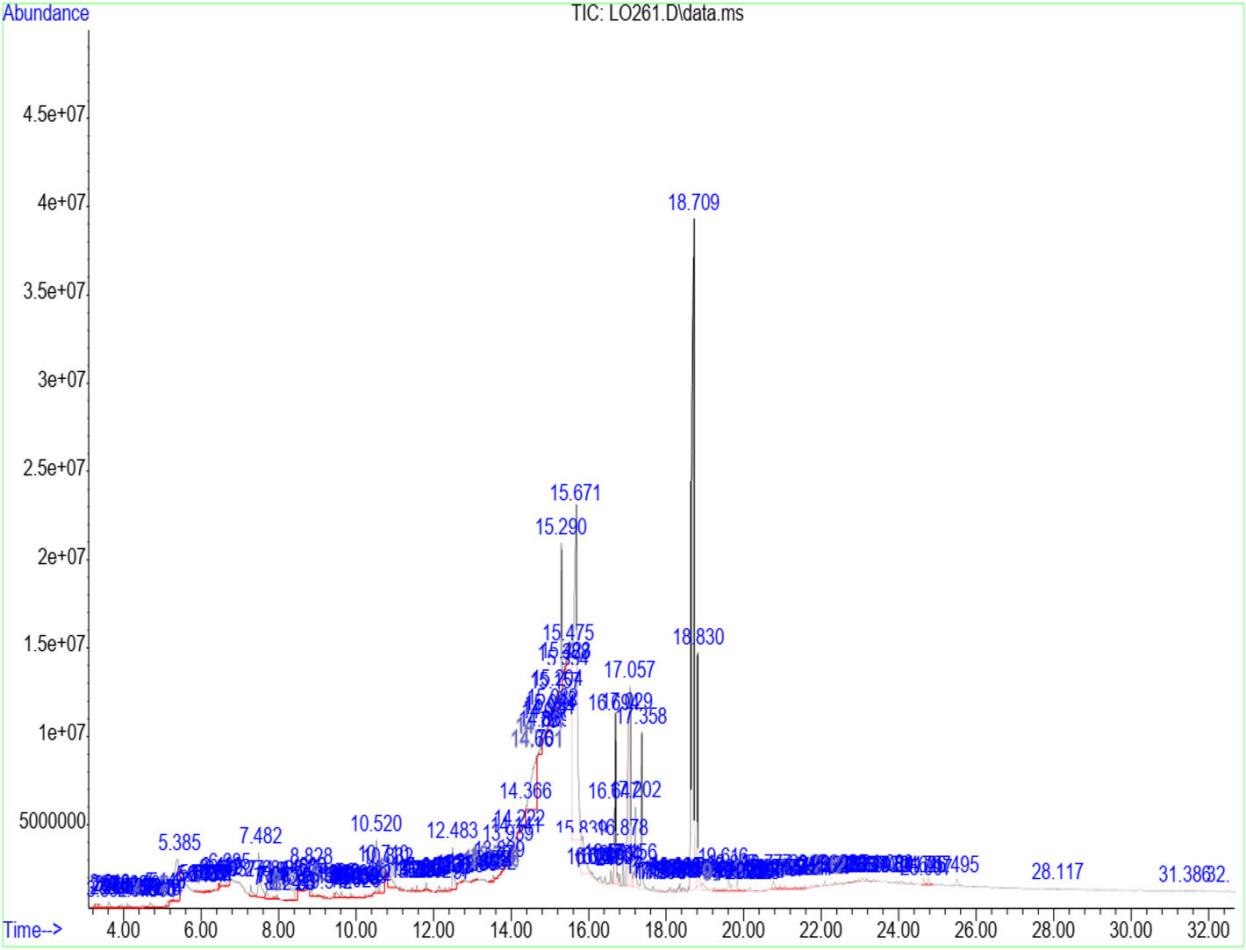
GC/MS chromatogram of fungal extract LO 261

**Fig. 7:**
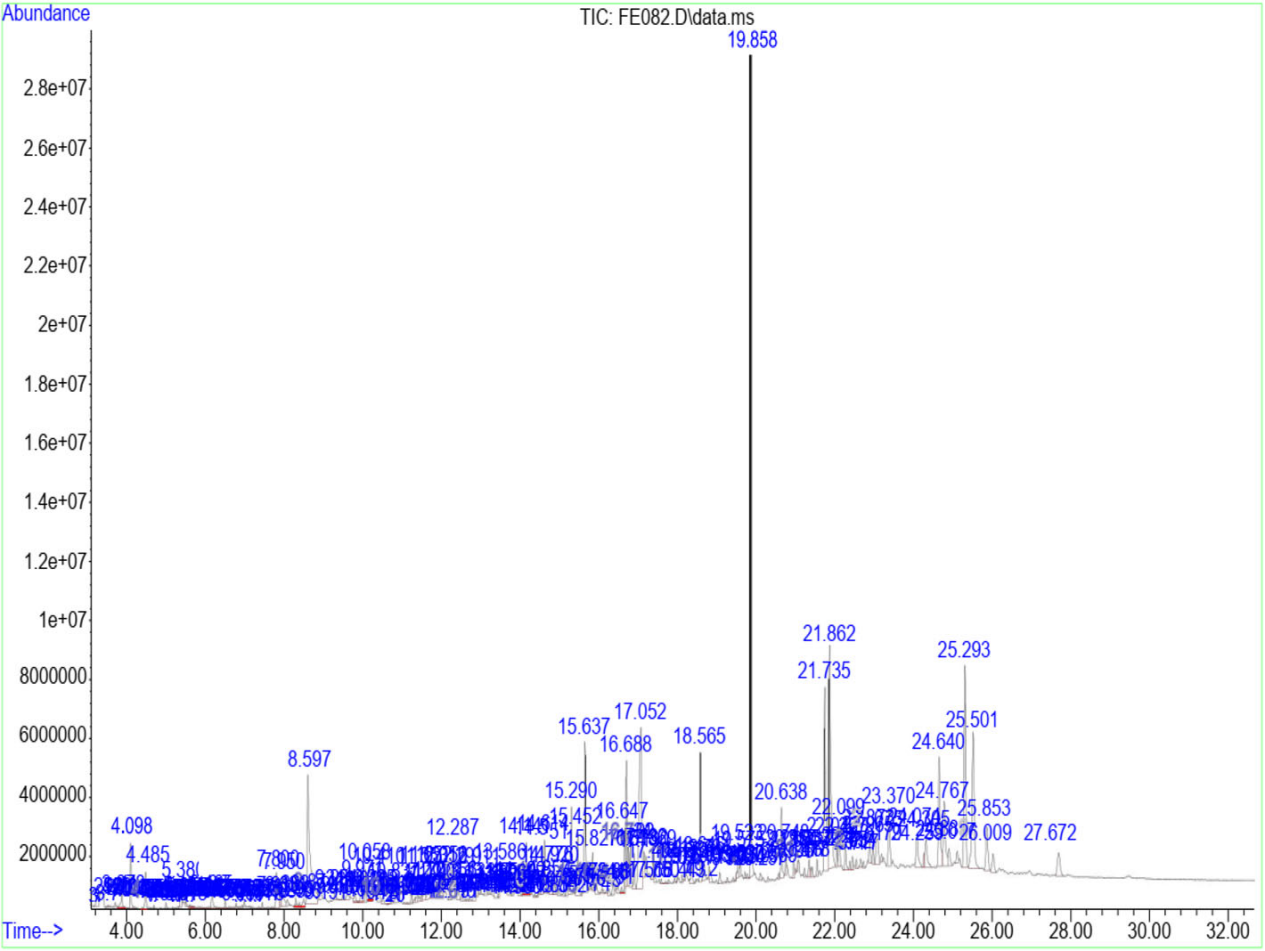
GC/MS chromatogram of fungal extract FE 082

**Fig. 8:**
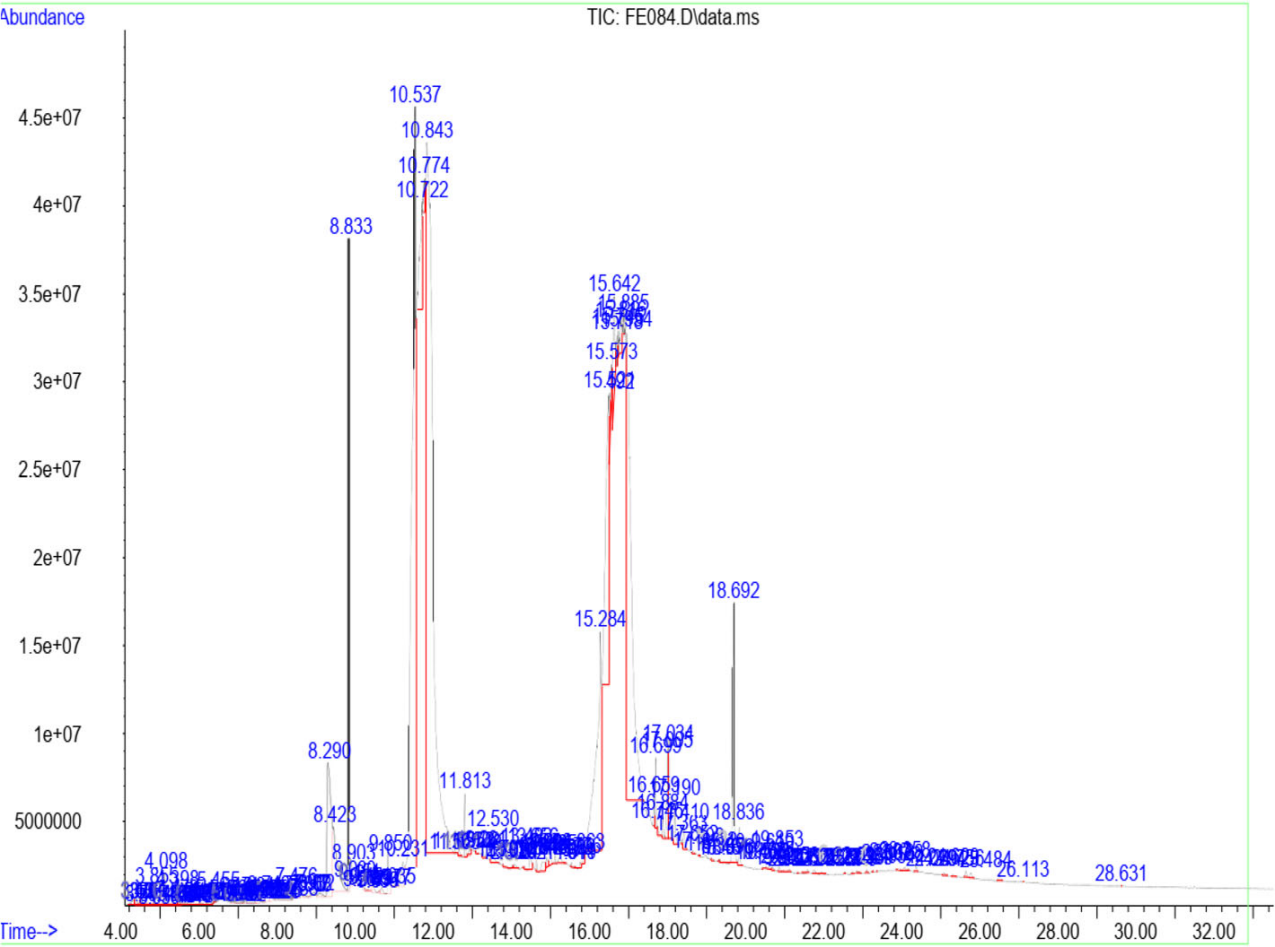
GC/MS chromatogram of fungal extract FE 084

**Fig. 9:**
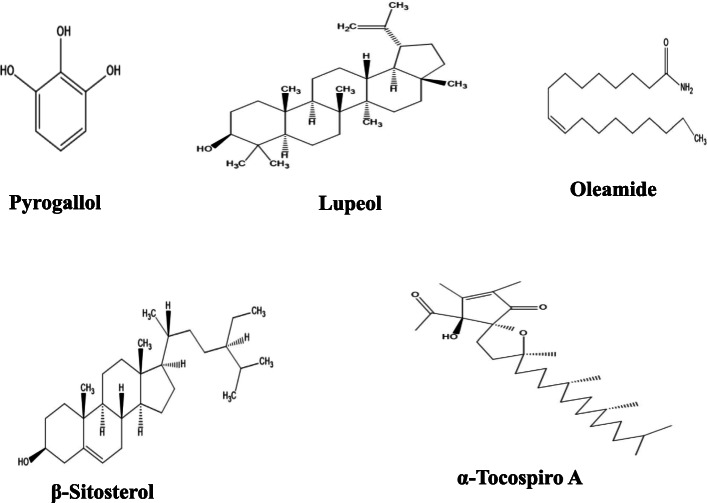
Chemical structures of representative of phenolic (pyrogallol), terpenoid (lupeol), fatty acid (oleamide), sterol (β-sitosterol), and tocopherol (α-tocospiro A) compounds present in extracts of the isolated endophytic fungi

Table 3 shows the retention time, molecular weight, molecular formula, and class of identified chemical constituents present in endophytic fungal extracts ZA163, MO211, LO 261, FE 082, and FE 084 with a substantial percentage compositions and quality match ranging from 86 to 99%. The chromatograms are shown in Figs. 4, 5, 6, 7, 8. The identified compounds include phenol and tocopherol (phenol, 2-methoxy; phenol, 2,6-dimethoxy-; phenol, 2,6-dimethoxy-4-(2-propenyl); pyrogallol; phenol, 2,2′-methylenebis [6-(1,1-dimethylethyl)-4-methyl; alpha tocospiro A; alpha tocospiro B, dl-alpha-tocopherol; and gamma tocopherol); terpenoids (phytol; lup-20(29)-en-3-one; lupeol; and urs-12-en-24-oic acid, 3-oxo-, methyl ester); sterols (campesterol; stigmasterol; and sitosterol); and fatty acids and its amide derivative (methyl stearate; oleic acid; linoleic acid; palmitic acid; and oleamide). Figure 9 shows the chemical structures of pyrogallol (phenolic), lupeol (terpenoid), and β-sitosterol (sterol) as representatives of the major classes of secondary metabolites as well as oleamide (amide derivative of fatty acid) and α-tocospiro A (tocopherol) present in the investigated endophytic fungi extracts.

**Table 3:** Identified chemical constituents of bioactive fungal extracts

S/N	Name of compound/ Molecular weight (g/mol)/ Class of identified compound	Molecular formular	RT (min)	Percent composition				
				ZA163	MO211	LO261	FE082	FE084
1.	Phenol, 2-methoxy- (124.14) Phenolic	C_7_H_8_O_2_	6.73	–	0.46	–	–	–
2.	Phenol, 2,6-dimethoxy- (154.16) Phenolic	C_8_H_10_O_3_	9.88	–	0.54	–	–	–
3.	1,2,3-Benzenetriol / pyrogallol (126.11) Phenolic	C_6_H_6_O_3_	10.84	10.72	19.84	1.46	–	31.33
4.	Phenol, 2,6-dimethoxy-4-(2-propenyl)- (194.23) Phenolic	C_11_H_14_O_3_	12.48	0.67	–	0.50	–	–
5.	Naphthalene, 1,6-dimethyl-4-(1-methylethyl)- (198.30) Phenolic	C_15_H_18_	13.19	–	0.52	–	–	–
6.	n-Hexadecanoic acid / palmitic acid (256.42) Fatty acid	C_16_H_32_O_2_	15.64	–	0.71	–	2.69	–
7.	9-Octadecenoic acid (Z)-, methyl ester (296.49) Fatty acid	C_19_H_36_O_2_	16.69	1.56	1.48	1.71	2.82	0.62
8.	Phytol (296.54) Terpenoid	C_20_H_40_O	16.79	–	–	–	1.09	–
9.	Methyl stearate (298.50) Fatty acid	C_19_H_38_O_2_	16.88	0.56	0.26	0.41	–	–
10.	Oleic acid (282.46) Fatty acid	C_18_H_34_O_2_	17.28	7.26	9.28	–	–	0.93
11.	9,12-Octadecadienoic acid (Z, Z)-, linoleic acid/linoelaidic (280.45) Fatty acid	C_18_H_32_O_2_	17.72	0.40	0.37	0.05	0.07	–
12.	9-Octadecenamide, (Z)- / oleamide (281.48) Fatty acid	C_18_H_35_NO	18.69	1.83	–	11.04	–	1.12
13.	Phenol, 2,2′-methylenebis[6-(1,1-dimethylethyl)-4-methyl (340.50) Phenolic	C_23_H_32_O_2_	19.05	–	–	–	0.19	–
14.	Alpha-tocospiro A / B (462.71) Tocopherol	C_29_H_50_O_4_	21.86	0.62	–	–	4.92	0.86
15.	DL-alpha-tocopherol / gamma tocopherol (430.71) Tocopherol	C_29_H_50_O_2_	23.37	0.15	0.06	–	1.47	–
16.	Campesterol (400.68) Sterol	C_28_H_48_O	24.07	–	0.09	–	–	0.93
17	Stigmasterol (412.69) Sterol	C_29_H_48_O	24.64	0.45	0.13	–	0.94	–
18.	Gamma/ beta. -Sitosterol (414.71) Sterol	C_29_H_50_O	24.77	0.42	0.14	–	1.79	–
19	Lup-20 (29)-en-3-one (424.70) Terpenoid	C_30_H_48_O	25.29	0.26	–	–	4.04	–
20	Lupeol (426.72) Terpenoid	C_30_H_50_O	25.50	–	–	–	3.41	–
21.	Urs-12-en-24-oic acid, 3-oxo-, methyl ester, (+)- (468.70) Terpenoid	C_31_H_48_O_3_	26.45	–	0.15	–	–	–

Fourteen compounds (0.06 to 19.84% percent composition) were identified in fungal extract MO211. Pyrogallol was recorded as the most abundant compound with percent composition of 19.84% while dl-alpha-tocopherol (0.06%) was the least identified compound. A total of twelve compounds were identified in ZA163 fungal extract, pyrogallol (10.72%) was the most abundant compound while dl-alpha-tocopherol (0.15%) was least identified. This was followed by fungal extract FE 082 with eleven identified compounds. Alpha tocospiro (4.92%) appeared as the most abundant while linoleic acid (0.07%) was the least identified compound. Fungal extracts FE 084 and LO 261 recorded six compounds each with pyrogallol (31.33%) and 9-octadecenamide (11.04%) recorded as most abundant compounds and 9-octadecenoic acid (Z)-, methyl ester (0.62%) and linoleic acid (0.05%) as least identified compounds respectively (Table 3).

## Discussion

In this study, eighteen endophytic fungi were successfully isolated from surface sterilized fresh leaf samples of eight medicinal plants. To the best of our knowledge, this is the first report describing the isolation of endophytic fungi residing in the leaf cells of the selected plant samples. After size separation by agarose gel electrophoresis of the ITS PCR products, the PCR products of the plant samples exhibited DNA band size range of 400–700 bp. Detailed taxonomic assignment of fungal isolates remain unresolved due to limitations inherent in fungal ITS sequences. The results obtained from this study thus confirmed that leaves of the medicinal plants: *Acalypha ornata*, *Albizia zygia*, *Alchornea cordifolia Chrysophyllum albidum*, *Ficus exasperata*, *Gomphrena celosioides*, *Millettia thonningii*, and *Newbouldia laevis* are host to endophytic fungi.

The crude fungal extracts showed vary degrees of antioxidant activity in the DPPH radical scavenging and reduction of ferric ion assays. DPPH (2, 2-diphenyl-1-picryl-hydrazyl) is a stable free radical that produces purple color in methanol. Antioxidant activity of the fungal extracts was measured by discoloration to yellow color following the formation of non-radical (2,2-diphenyl-1-hydrazine) molecule [17]. Fungal extracts ZA163 and MO211 exhibited significant (*p* < 0.05) DPPH radical scavenging activity.

The metal chelating capacity of a compound may also serve as a significant indicator of its potential antioxidant activity [35]. The iron chelating activity of all fungal extracts was determined by reaction with *ortho*-phenanthroline. Fe^3+^ is reduced to Fe^2+^ by an antioxidant and the formed Fe^2+^ rapidly react with phenanthroline to form a stable red orange colored complex [33, 36]. Isolated fungal extracts ZA 163, LO 261, CA 041, CA 042, CA 043, FE 082, FE 084, and GE 091 from *A. zygia*, *A. cordifolia*, *C. albidum*, *F. exasperata*, and *G. celosioides* medicinal plants demonstrated iron chelating capability. Similar observations of antioxidant activity of endophytic fungi isolates have been reported in some medicinal plants such as *Bauhinia racemosa*, *Distylium chinense*, *Euphorbia hirta*, *Guazoma tomentosa*, *Phoenix dactylifer*, *Phyllanthus amarus*, and *Senna spectabilis* [4, 37–41]. Our results suggest that endophytic fungi that reside in the leaves of *A. ornata*, *A. zygia*, *A. cordifolia*, *C. albidum*, *F. exasperata*, *G. celosioides*, *M. thonningii*, and *N. laevis* showed promising antioxidant activity.

Fungal extracts ZA163, MO211, LO 261, FE 082, and FE 084 were among the fungal extracts that exhibited effective antioxidant activity with a substantial yield of extract, thus were selected for phytochemical analysis via GC/MS analytical technique. The identified compounds represented phenolic, terpenoid, and sterol classes of secondary metabolites as well as tocopherols and fatty acids. Most of these chemical constituents have been reported to demonstrate remarkable antioxidant activity. Phenolic compounds act as natural antioxidants that exert therapeutic effects such as anti-inflammatory, antidiabetic, antimicrobial, antiviral and vasodilatory effects and prevent various forms of diseases [42–44]. Tocopherol plays an important role as a lipid antioxidant in stabilizing subcellular membranes [45]. Fatty acids are able to reduce oxidative stress from free radicals by exerting an antioxidant role [46, 47]. Pyrogallol (phenol), alpha tocospiro (tocopherol), and oleamide (amide derivative of fatty acid) are present as the major components in the investigated endophytic fungi extracts. Previous studies have reported the isolation and characterization of graphislactone A, a phenolic benzopyranone antioxidant compound from the endophytic fungus *Cephalosporium sp*. inhabiting the medicinal plant *Trachelospermum jasminoides* [11]. Similarly, isolation and characterization of pestacin and isopestacin, a coumarone antioxidant and antifungal agents from the endophytic fungus *Pestalotiopsis microspora* that resides in the medicinal plant *Terminalia morobensis* was reported in literature [12, 13]. These results suggest that these compounds and others identified in fungal extracts ZA163 from *Albizia zygia*, MO211 from *Millettia thonningii*, LO 261 from *Alchornea cordifolia*, FE 082 and FE 084 from *Ficus exasperata* could be responsible for the antioxidant activity demonstrated by the endophytes.

## Conclusion

The results obtained from this study established that endophytic fungi isolated from medicinal plants: *Acalypha ornata*, *Albizia zygia*, *Alchornea cordifolia*, *Chrysophyllum albidum*, *Ficus exasperata*, *Gomphrena celosioides*, *Millettia thonningii*, and *Newbouldia laevis* commonly used in Nigeria local herbal remedies may be a potential source of bioactive compounds, which may be used for the development of antioxidant drugs. Phytochemical analysis revealed the presence of phenols, tocopherols, sterols, terpenoids, and fatty acids that may have contributed to the observed effect. These findings indicate that endophytic fungi hold promise for large scale production of free radical scavenging agents, which can be developed as drug molecules used in the intervention of oxidative stress-mediated diseases.

## Data Availability

The datasets generated and/or analyzed during the present study are available from the corresponding author on reasonable request.

## Abbreviations

PDA: Potato dextrose agar
ITS: Internal transcribed spacer region
DNA: Deoxyribonucleic acid
PDB: Potato dextrose broth
PCR: Polymerase chain reaction
dNTP: Deoxynucleoside triphosphate
NCBI: National Centre for Biotechnology Information
BLAST: Basic Local Alignment Search Tool
DPPH: 2, 2-diphenyl-1-picryl-hydrazyl
UV-vis: Ultraviolet-visible
GC/MS: Gas chromatography/mass spectrometry
*O*-phenanthroline: *Ortho*-phenanthroline
IC_50_: Half maximal inhibitory concentration
SEM: Standard error of mean
